# Supplementary material for: Whole blood transcriptome biomarkers of unruptured intracranial aneurysm
Source: PLoS One. 2020 Nov 6;15(11):e0241838. doi: 10.1371/journal.pone.0241838 (PMC7647097; doi:10.1371/journal.pone.0241838)
Supplement: S1 Table — (DOCX) [file pone.0241838.s001.docx]

**S1 Table**. **Characteristics of 27 aneurysms in all patients with intracranial aneurysms (6 patients had multiple intracranial aneurysms).***

| **ID** | **IA Size (mm)** | **IA Location** | **Presence of Additional IAs** | **Family History of IA** |
| --- | --- | --- | --- | --- |
| ***Training Cohort*** | | | | |
| A1 | 10 | ICA | No | No |
| A2 | 19 | ICA | No | No |
| A3 | 9.3 | VB Junction | No | No |
| A4 | 3.2 | MCA | No | No |
| A5 | 7 | MCA | No | No |
| A6 | 5 | ACom | No | No |
| A7 | 2.7 | ICA | No | Yes |
| A8 | 2.9 | SCA | No | No |
| A9 | 10 | MCA | No | No |
| A10 | 2 | MCA | No | No |
| A11 | 7 | ICA | +1 (7 mm ICA) | No |
| A12 | 3.1 | ACom | No | No |
| A13 | 2.5 | SHA | No | No |
| A14 | 3 | ICA | No | No |
| A15 | 5.1 | ICA | No | No |
| A16 | 4.85 | ICA | No | No |
| A17 | 3.7 | ACom | No | No |
| A18 | 1.4 | MCA | No | No |
| A19 | 2.8 | SHA | No | No |
| A20 | 2 | ACA | No | No |
| A21 | 3.9 | BT | No | No |
| A22 | 8 | MCA | No | No |
| A23 | 1 | ICA | No | No |
| A24 | 3 | MCA | No | No |
| ***Testing Cohort*** | | | | |
| A25 | 7.2 | ACom | +1 (1.5 mm ICA) | No |
| A26 | 8 | SHA | No | No |
| A27 | 13 | ICA | No | No |
| A28 | 3 | ICA | No | No |
| A29 | 5.5 | ACA | +2 (5 mm ICA, 2 mm PCom) | No |
| A30 | 7 | MCA | +1 (3.5 mm ACA) | No |
| A31 | 2 | ICA | No | No |
| A32 | 10 | ICA | +2 (8 mm ICA, 2 mm PCom) | No |
| A33 | 2.8 | ICA | No | No |
| A34 | 3 | ACA | +1 (2 mm BT) | No |

*Intracranial aneurysm (IA) size ranged from 1 mm to 19 mm in greatest diameter. The aneurysms were situated at various locations in the circle of Willis, with most around the internal carotid artery (ICA) and its branches. Three patients with IAs had a family history of the disease. In general, digital subtraction angiography was performed for confirmation of IA presence after an incidental finding of IA on noninvasive imaging or for follow-up imaging of a previously detected IA. ACA=anterior cerebral artery, ACom=anterior communicating artery, BT=basilar terminus, CTA=computed tomography angiography, DSA=digital subtraction angiography, IA=intracranial aneurysm, ICA=internal carotid artery, MCA=middle cerebral artery, MRA=magnetic resonance angiography, MRI=magnetic resonance imaging, OA=ophthalmic artery, PCom=posterior communicating artery, SCA=superior cerebellar artery, SHA=superior hypophyseal artery, VB=vertebrobasilar.
