# Supplementary material for: Whole blood transcriptome biomarkers of unruptured intracranial aneurysm
Source: PLoS One. 2020 Nov 6;15(11):e0241838. doi: 10.1371/journal.pone.0241838 (PMC7647097; doi:10.1371/journal.pone.0241838)
Supplement: S2 Table — (DOCX) [file pone.0241838.s002.docx]

**S2 Table. Cohort assignment and RNA quality.***

| **Sample ID** | **Class** | **260/280** | **RIN** | **M Seqs** | **Seq Length** | **%GC** | **%Aligned** |
| --- | --- | --- | --- | --- | --- | --- | --- |
| ***Training Cohort*** | | | | | | | |
| C1 | Control | 1.97 | 9.5 | 52.8 | 51 | 48 | 96.60% |
| C2 | Control | 2.01 | 7.7 | 69.5 | 51 | 45 | 95.30% |
| C3 | Control | 1.91 | 10.0 | 67 | 51 | 44 | 97.00% |
| C4 | Control | 1.95 | 10.0 | 64 | 51 | 44 | 97.00% |
| C5 | Control | 2.02 | 9.2 | 50.5 | 51 | 49 | 96.50% |
| C6 | Control | 2.02 | 8.8 | 30.2 | 51 | 48 | 96.20% |
| C7 | Control | 1.99 | 9.2 | 60.9 | 51 | 46 | 95.80% |
| C8 | Control | 2.03 | 8.9 | 46.6 | 51 | 49 | 95.70% |
| C9 | Control | 2.01 | 9.3 | 75.6 | 51 | 46 | 96.30% |
| C10 | Control | 2.01 | 9.3 | 55.5 | 51 | 46 | 95.80% |
| C11 | Control | 2.01 | 7.5 | 55.6 | 51 | 53 | 97.00% |
| C12 | Control | 1.96 | 9.2 | 52.4 | 51 | 47 | 96.20% |
| C13 | Control | 1.96 | 8.4 | 34.7 | 51 | 47 | 95.20% |
| C14 | Control | 1.85 | 8.7 | 54.1 | 51 | 45 | 95.20% |
| C15 | Control | 1.93 | 7.9 | 65.9 | 51 | 44 | 96.00% |
| C16 | Control | 2.03 | 7.3 | 69 | 51 | 46 | 96.10% |
| C17 | Control | 2.01 | 7.2 | 57.6 | 51 | 45 | 95.70% |
| C18 | Control | 2.01 | 8.4 | 60.7 | 51 | 47 | 95.90% |
| C19 | Control | 2.05 | 8.6 | 52.9 | 51 | 51 | 96.40% |
| C20 | Control | 2.15 | 9.7 | 64.4 | 51 | 48 | 96.50% |
| C21 | Control | 1.98 | 7.8 | 57.2 | 51 | 45 | 96.80% |
| C22 | Control | 1.95 | 9.0 | 58.2 | 51 | 49 | 95.90% |
| C23 | Control | 2.02 | 9.5 | 35.7 | 51 | 47 | 96.50% |
| A1 | Aneurysm | 2.00 | 8.8 | 53.6 | 51 | 50 | 95.70% |
| A2 | Aneurysm | 1.85 | 8.2 | 49.3 | 51 | 46 | 95.90% |
| A3 | Aneurysm | 2.03 | 9.5 | 50.8 | 51 | 49 | 96.50% |
| A4 | Aneurysm | 1.98 | 9.5 | 52.2 | 51 | 49 | 96.50% |
| A5 | Aneurysm | 1.66 | 7.4 | 36.3 | 51 | 47 | 95.80% |
| A6 | Aneurysm | 1.87 | 6.7 | 65.6 | 51 | 51 | 98.00% |
| A7 | Aneurysm | 1.93 | 6.5 | 51.4 | 51 | 45 | 96.80% |
| A8 | Aneurysm | 2.04 | 9.4 | 54.9 | 51 | 48 | 96.70% |
| A9 | Aneurysm | 2.06 | 8.9 | 66.3 | 51 | 44 | 96.80% |
| A10 | Aneurysm | 2.01 | 9.8 | 44.7 | 51 | 46 | 96.40% |
| A11 | Aneurysm | 1.92 | 7.6 | 44.2 | 51 | 46 | 96.20% |
| A12 | Aneurysm | 1.92 | 6.0 | 56.5 | 51 | 45 | 95.10% |
| A13 | Aneurysm | 1.95 | 6.8 | 47.7 | 51 | 44 | 96.20% |
| A14 | Aneurysm | 1.97 | 9.2 | 49 | 51 | 49 | 95.50% |
| A15 | Aneurysm | 2.04 | 8.9 | 46.2 | 51 | 47 | 96.40% |
| A16 | Aneurysm | 2.02 | 7.2 | 69.6 | 51 | 44 | 96.10% |
| A17 | Aneurysm | 1.95 | 6.1 | 70.5 | 51 | 48 | 97.40% |
| A18 | Aneurysm | 1.96 | 8.9 | 71.3 | 51 | 47 | 97.20% |
| A19 | Aneurysm | 1.96 | 7.8 | 59.3 | 51 | 44 | 95.40% |
| A20 | Aneurysm | 2.03 | 9.4 | 53.3 | 51 | 49 | 95.60% |
| A21 | Aneurysm | 2.03 | 9.1 | 66.2 | 51 | 48 | 96.40% |
| A22 | Aneurysm | 2.07 | 8.6 | 69.4 | 51 | 47 | 96.60% |
| A23 | Aneurysm | 1.98 | 8.1 | 66.3 | 51 | 45 | 96.80% |
| A24 | Aneurysm | 2.01 | 8.8 | 66.6 | 51 | 47 | 96.00% |
| ***Testing Cohort*** | | | | | | |  |
| C24 | Control | 1.83 | 9.3 | 72.1 | 51 | 48 | 96.10% |
| C25 | Control | 2.05 | 8.1 | 52.4 | 51 | 50 | 95.80% |
| C26 | Control | 2.02 | 8.5 | 63.6 | 51 | 47 | 96.30% |
| C27 | Control | 1.97 | 9.1 | 67.9 | 51 | 46 | 96.50% |
| C28 | Control | 2.15 | 8.5 | 49.1 | 51 | 48 | 95.40% |
| C29 | Control | 1.99 | 8.8 | 62 | 51 | 44 | 96.30% |
| C30 | Control | 1.95 | 7.8 | 60.6 | 51 | 43 | 96.30% |
| C31 | Control | 1.97 | 8.6 | 76.2 | 51 | 49 | 96.40% |
| C32 | Control | 1.98 | 9.0 | 64.8 | 51 | 49 | 96.50% |
| C33 | Control | 1.80 | 7.8 | 52.5 | 51 | 44 | 96.70% |
| A25 | Aneurysm | 2.04 | 10.0 | 44.1 | 51 | 46 | 96.00% |
| A26 | Aneurysm | 2.00 | 8.5 | 71.9 | 51 | 48 | 96.50% |
| A27 | Aneurysm | 2.00 | 8.5 | 71.9 | 51 | 49 | 96.30% |
| A28 | Aneurysm | 2.01 | 9.6 | 62.9 | 51 | 46 | 95.60% |
| A29 | Aneurysm | 1.98 | 9.6 | 49.3 | 51 | 47 | 95.00% |
| A30 | Aneurysm | 1.96 | 6.1 | 33 | 51 | 45 | 96.20% |
| A31 | Aneurysm | 2.06 | 9.0 | 51.3 | 51 | 48 | 96.20% |
| A32 | Aneurysm | 2.02 | 9.0 | 59.9 | 51 | 49 | 96.10% |
| A33 | Aneurysm | 1.97 | 7.9 | 51 | 51 | 49 | 96.60% |
| A34 | Aneurysm | 2.04 | 9.4 | 69.6 | 51 | 48 | 96.30% |

*RIN=RNA integrity number, M=millions, Seq=sequences.
