# Supplementary material for: Whole blood transcriptome biomarkers of unruptured intracranial aneurysm
Source: PLoS One. 2020 Nov 6;15(11):e0241838. doi: 10.1371/journal.pone.0241838 (PMC7647097; doi:10.1371/journal.pone.0241838)
Supplement: S3 Table — (DOCX) [file pone.0241838.s003.docx]

**S3 Table. Per-gene performance of the 18 model transcripts.***

|  | **Testing** | | | **Testing** | | |
| --- | --- | --- | --- | --- | --- | --- |
| **Gene** | **Acc** | **Sens** | **Spec** | **Acc** | **Sens** | **Spec** |
| *ATF3* | 0.34 | 0.25 | 0.43 | 0.50 | 1.00 | 0.00 |
| *CBWD6* | 0.68 | 0.83 | 0.52 | 0.40 | 0.70 | 0.10 |
| *CCDC85B*† | 0.72 | 0.79 | 0.65 | 0.85 | 1.00 | 0.70 |
| *CCR8*† | 0.70 | 0.75 | 0.65 | 0.70 | 0.60 | 0.80 |
| *CHMP4B* | 0.55 | 1.00 | 0.09 | 0.45 | 0.90 | 0.00 |
| *CLEC4F* | 0.32 | 0.25 | 0.39 | 0.40 | 0.80 | 0.00 |
| *CXCL10* | 0.34 | 0.25 | 0.43 | 0.45 | 0.90 | 0.00 |
| *FN1* | 0.34 | 0.25 | 0.43 | 0.50 | 0.00 | 1.00 |
| *MT2A* | 0.32 | 0.29 | 0.35 | 0.50 | 1.00 | 0.00 |
| *MZT2B* | 0.72 | 0.75 | 0.70 | 0.60 | 0.70 | 0.50 |
| *PCSK1N*† | 0.77 | 0.92 | 0.61 | 0.85 | 0.90 | 0.80 |
| *PIM3* | 0.66 | 0.67 | 0.65 | 0.75 | 0.90 | 0.60 |
| *SLC37A3* | 0.60 | 0.67 | 0.52 | 0.40 | 0.50 | 0.30 |
| *ST6GALNAC1* | 0.68 | 0.58 | 0.78 | 0.55 | 0.30 | 0.80 |
| *TCN2* | 0.49 | 0.88 | 0.09 | 0.55 | 1.00 | 0.10 |
| *TIFAB* | 0.36 | 0.67 | 0.04 | 0.60 | 1.00 | 0.20 |
| *TNFRSF4*† | 0.74 | 0.92 | 0.57 | 0.85 | 0.90 | 0.80 |
| *UFSP1* | 0.74 | 0.83 | 0.65 | 0.60 | 1.00 | 0.20 |

*Acc=accuracy, sens=sensitivity, spec=specificity.

†Transcripts with accuracy ≥0.70 in both cohorts.
