# Supplementary material for: Whole blood transcriptome biomarkers of unruptured intracranial aneurysm
Source: PLoS One. 2020 Nov 6;15(11):e0241838. doi: 10.1371/journal.pone.0241838 (PMC7647097; doi:10.1371/journal.pone.0241838)
Supplement: S4 Table — (DOCX) [file pone.0241838.s004.docx]

**S4 Table. Transcripts in the 2 significant networks constructed by Ingenuity Pathway Analysis (IPA).***

| **Network** | **Molecules in Network** | **P-score** | **Focus Molecules** | **Top Diseases and Functions** |
| --- | --- | --- | --- | --- |
| A  *LASSO Network 1* | ADGRG3, Akt, ANGPTL1, Ap1, ATF, **ATF3**, **CCR8**, CD3, CLEC2A, Creb, **CXCL10**, Eriodictyol, ERK, ERK1/2, **FN1**, Gm9573, GPR171, IFN Beta, IgG, IL1, Immunoglobulin, Jnk, MIR320, **MT2A**, NFkB (complex), P38 MAPK, **PCSK1N**, PI3K (complex), **PIM3**, Pka, RNU1-1, TCR, Tnf (family), **TNFRSF4**, Vegf | 20 | 8 | Cardiovascular System Development and Function, Cell Death and Survival, Tissue Development |
| B  *LASSO Network 2* | AR, C9, **CBWD3/CBWD6**, **CCDC85B**, **CHMP4B**, CLU, CNN2, CTNNB1, ENO3, FBLN2, FERMT2, FZD8, ITGB7, KNTC1, LIPF, mir-192, MMP16, MYL4, **MZT2B**, NUDT5, RGS6, SERPINA5, SETD7, **SLC37A3**, SLC5A3, ST13, **ST6GALNAC1**, **TCN2**, TERC, TNFAIP2, TNS1, TP53, WNT5B, ZBTB7A, ZNF778 | 17 | 7 | Cancer, Endocrine System Disorders, Gastrointestinal Disease |

*Transcripts in bold are part of the LASSO gene panel.
