# Supplementary material for: Whole blood transcriptome biomarkers of unruptured intracranial aneurysm
Source: PLoS One. 2020 Nov 6;15(11):e0241838. doi: 10.1371/journal.pone.0241838 (PMC7647097; doi:10.1371/journal.pone.0241838)
Supplement: S5 Table — (DOCX) [file pone.0241838.s005.docx]

**S5 Table. Significant disease and biological functions assigned by Ingenuity Pathway Analysis for genes identified by LASSO (Benjamini-Hochberg p-value<0.05, at least 3 molecules assigned).**

| Categories | Diseases or Functions Annotation | B-H p-value | Molecules |
| --- | --- | --- | --- |
| Cancer, Cell Death and Survival, Organismal Injury and Abnormalities, Tumor Morphology | Cell death of tumor cells | 0.0349 | ATF3, CHMP4B, PIM3 |
| Cancer, Cellular Development, Cellular Growth and Proliferation, Organismal Injury and Abnormalities, Tumor Morphology | Proliferation of tumor cells | 0.0356 | ATF3, PIM3, TNFRSF4 |
| Cancer, Gastrointestinal Disease, Hepatic System Disease, Organismal Injury and Abnormalities | Hepatocellular carcinoma | 0.0392 | CXCL10, FN1, MT2A, PIM3 |
| Cancer, Organismal Injury and Abnormalities | Carcinoma | 0.0356 | ATF3, CBWD3/CBWD6, CCDC85B, CCR8, CHMP4B, CLEC4F, CXCL10, FN1, MT2A, MZT2B, PCSK1N, PIM3, SLC37A3, ST6GALNAC1, TCN2, TIFAB, TNFRSF4, UFSP1 |
|  | Metastasis of tumor cell lines | 0.0193 | ATF3, CCR8, FN1 |
|  | Invasive carcinoma | 0.0252 | ATF3, CXCL10, FN1 |
| Cancer, Organismal Injury and Abnormalities, Reproductive System Disease | Ductal breast carcinoma | 0.0239 | CXCL10, FN1, MT2A |
| Cardiovascular System Development and Function, Organismal Development | Vasculogenesis | 0.0343 | ATF3, CXCL10, FN1, PIM3 |
| Cell Cycle | Mitosis | 0.0395 | CHMP4B, CXCL10, PIM3 |
| Cell Death and Survival | Necrosis | 0.0193 | ATF3, CCR8, CHMP4B, CXCL10, FN1, MT2A, PCSK1N, PIM3, TNFRSF4 |
|  | Cell viability | 0.0224 | ATF3, CXCL10, FN1, PCSK1N, PIM3, TNFRSF4 |
|  | Cell death of immune cells | 0.0174 | ATF3, CCR8, CXCL10, FN1, TNFRSF4 |
|  | Cell viability of lung cancer cell lines | 0.0174 | ATF3, FN1, PIM3 |
|  | Cell death of T lymphocytes | 0.0213 | CCR8, CXCL10, TNFRSF4 |
|  | Apoptosis of leukocytes | 0.0353 | ATF3, CCR8, TNFRSF4 |
| Cell Morphology | Sprouting | 0.0178 | ATF3, FN1, MT2A, PIM3 |
| Cell Morphology, Cellular Development | Branching of cells | 0.0178 | ATF3, FN1, MT2A, PIM3 |
| Cell Signaling, Cellular Function and Maintenance, Molecular Transport, Vitamin and Mineral Metabolism | Flux of Ca2+ | 0.0201 | CCR8, CXCL10, FN1 |
| Cell Signaling, Molecular Transport, Vitamin and Mineral Metabolism | Quantity of Ca2+ | 0.0288 | CCR8, CXCL10, FN1 |
| Cell-mediated Immune Response, Cellular Movement, Hematological System Development and Function, Immune Cell Trafficking | Cell movement of T lymphocytes | 0.0174 | CCR8, CXCL10, FN1, TNFRSF4 |
| Cell-To-Cell Signaling and Interaction | Adhesion of tumor cell lines | 0.0213 | CXCL10, FN1, ST6GALNAC1 |
| Cell-To-Cell Signaling and Interaction, Cellular Movement, Hematological System Development and Function, Immune Cell Trafficking | Recruitment of leukocytes | 0.0223 | CCR8, CXCL10, FN1 |
| Cell-To-Cell Signaling and Interaction, Hematological System Development and Function, Immune Cell Trafficking, Inflammatory Response | Activation of leukocytes | 0.0252 | ATF3, CXCL10, FN1, TNFRSF4 |
|  | Activation of macrophages | 0.0192 | ATF3, CXCL10, FN1 |
|  | Activation of T lymphocytes | 0.0237 | CXCL10, FN1, TNFRSF4 |
| Cellular Assembly and Organization | Quantity of filaments | 0.0174 | ATF3, FN1, MT2A |
| Cellular Development, Cellular Growth and Proliferation | Cell proliferation of tumor cell lines | 0.0378 | ATF3, CXCL10, FN1, MT2A, TCN2, TNFRSF4 |
|  | Proliferation of blood cells | 0.0403 | CXCL10, FN1, PIM3, TNFRSF4 |
| Cellular Development, Cellular Growth and Proliferation, Connective Tissue Development and Function | Proliferation of fibroblast cell lines | 0.0327 | CXCL10, FN1, PIM3 |
| Cellular Development, Cellular Growth and Proliferation, Nervous System Development and Function, Tissue Development | Growth of neurites | 0.0378 | FN1, MT2A, PCSK1N |
| Cellular Development, Connective Tissue Development and Function, Tissue Development | Differentiation of adipocytes | 0.0193 | ATF3, CCDC85B, FN1 |
| Cellular Development, Skeletal and Muscular System Development and Function, Tissue Development | Differentiation of muscle | 0.0223 | ATF3, CXCL10, FN1 |
| Cellular Function and Maintenance | Cellular homeostasis | 0.0319 | CCR8, CHMP4B, CXCL10, FN1, MT2A, TNFRSF4 |
|  | Ion homeostasis of cells | 0.0207 | CCR8, CXCL10, FN1, MT2A |
| Cellular Function and Maintenance, Hematological System Development and Function | Function of T lymphocytes | 0.0192 | ATF3, CXCL10, TNFRSF4 |
| Cellular Growth and Proliferation, Lymphoid Tissue Structure and Development | Proliferation of lymphatic system cells | 0.0353 | CXCL10, FN1, PIM3, TNFRSF4 |
| Cellular Movement | Cell movement of muscle cells | 0.0174 | ATF3, CCR8, CXCL10, FN1 |
|  | Migration of breast cancer cell lines | 0.0201 | ATF3, CXCL10, FN1 |
| Cellular Movement, Embryonic Development | Cell movement of embryonic cell lines | 0.0174 | CCR8, CXCL10, FN1 |
| Cellular Movement, Hair and Skin Development and Function | Cell movement of epithelial cell lines | 0.0174 | CCR8, CXCL10, FN1 |
| Cellular Movement, Hematological System Development and Function, Immune Cell Trafficking | Migration of antigen presenting cells | 0.0174 | CCR8, CXCL10, FN1 |
|  | Cellular infiltration by mononuclear leukocytes | 0.0178 | CXCL10, FN1, TNFRSF4 |
| Cellular Movement, Hematological System Development and Function, Immune Cell Trafficking, Inflammatory Response | Migration of phagocytes | 0.0201 | CCR8, CXCL10, FN1 |
| Cellular Movement, Renal and Urological System Development and Function | Cell movement of kidney cell lines | 0.0174 | CCR8, CXCL10, FN1 |
| Cellular Movement, Skeletal and Muscular System Development and Function | Cell movement of vascular smooth muscle cells | 0.0174 | ATF3, CCR8, FN1 |
| DNA Replication, Recombination, and Repair | Synthesis of DNA | 0.0289 | ATF3, CXCL10, FN1 |
| Endocrine System Disorders, Gastrointestinal Disease, Immunological Disease, Metabolic Disease, Organismal Injury and Abnormalities | Insulin-dependent diabetes mellitus | 0.034 | CCR8, CXCL10, TNFRSF4 |
| Endocrine System Disorders, Gastrointestinal Disease, Metabolic Disease, Organismal Injury and Abnormalities | Diabetes mellitus | 0.0178 | ATF3, CCR8, CXCL10, FN1, MT2A, TNFRSF4 |
| Gastrointestinal Disease, Inflammatory Disease | Inflammatory Bowel Disease | 0.0289 | CCR8, CXCL10, FN1 |
| Gastrointestinal Disease, Inflammatory Disease, Inflammatory Response, Organismal Injury and Abnormalities | Colitis | 0.0268 | CXCL10, FN1, TNFRSF4 |
| Gastrointestinal Disease, Inflammatory Response | Inflammation of gastrointestinal tract | 0.0192 | CCR8, CXCL10, FN1, TNFRSF4 |
| Hematological System Development and Function, Inflammatory Response, Tissue Morphology | Quantity of phagocytes | 0.034 | ATF3, CXCL10, TNFRSF4 |
| Hematological System Development and Function, Tissue Morphology | Quantity of myeloid cells | 0.0192 | ATF3, CXCL10, PIM3, TNFRSF4 |
| Immunological Disease | Systemic autoimmune syndrome | 0.0403 | CCR8, CXCL10, FN1, MT2A, TNFRSF4 |
| Immunological Disease, Inflammatory Disease, Inflammatory Response, Neurological Disease, Organismal Injury and Abnormalities | Experimental autoimmune encephalomyelitis | 0.0223 | CCR8, CXCL10, TNFRSF4 |
| Infectious Diseases | HIV infection | 0.0271 | CXCL10, FN1, MT2A, TNFRSF4 |
| Inflammatory Response | Inflammation of absolute anatomical region | 0.0356 | ATF3, CCR8, CXCL10, FN1, TNFRSF4 |
|  | Inflammatory response | 0.0349 | ATF3, CXCL10, FN1, TNFRSF4 |
| Inflammatory Response, Neurological Disease | Inflammation of central nervous system | 0.0178 | CCR8, CXCL10, FN1, TNFRSF4 |
| Inflammatory Response, Respiratory Disease | Inflammation of respiratory system component | 0.0223 | ATF3, CXCL10, FN1, TNFRSF4 |
| Lipid Metabolism, Small Molecule Biochemistry | Synthesis of lipid | 0.0356 | ATF3, CXCL10, FN1, ST6GALNAC1 |
| Molecular Transport | Transport of molecule | 0.032 | ATF3, CXCL10, FN1, MT2A, TCN2, TNFRSF4 |
