# Supplementary material for: Whole blood transcriptome biomarkers of unruptured intracranial aneurysm
Source: PLoS One. 2020 Nov 6;15(11):e0241838. doi: 10.1371/journal.pone.0241838 (PMC7647097; doi:10.1371/journal.pone.0241838)
Supplement: S6 Table — (DOCX) [file pone.0241838.s006.docx]

**S6 Table. The testing data.***

| **ID** | ***TNFRSF4*** | ***ATF3*** | ***CCDC85B*** | ***MT2A*** | ***ST6GALNAC1*** | ***CLEC4F*** | ***MZT2B*** | ***FN1*** | ***CHMP4B*** | ***TCN2*** | ***PIM3*** | ***CCR8*** | ***CXCL10*** | ***TIFAB*** | ***UFSP1*** | ***SLC37A3*** | ***CBWD7*** | ***PCSK1N*** |
| --- | --- | --- | --- | --- | --- | --- | --- | --- | --- | --- | --- | --- | --- | --- | --- | --- | --- | --- |
| C24 | 1.60 | 1.14 | 2.83 | 20.23 | 1.41 | 2.06 | 12.07 | 5.33 | 58.47 | 5.85 | 37.52 | 2.93 | 3.80 | 0.69 | 4.87 | 20.61 | 1.49 | 0.31 |
| C25 | 1.33 | 0.83 | 2.58 | 17.12 | 1.38 | 0.97 | 10.93 | 5.25 | 57.69 | 4.68 | 39.64 | 3.35 | 2.58 | 0.27 | 1.94 | 34.16 | 1.50 | 0.42 |
| C26 | 2.46 | 1.38 | 3.69 | 14.07 | 3.86 | 6.02 | 19.36 | 6.58 | 60.11 | 5.08 | 38.82 | 4.43 | 6.75 | 1.50 | 4.74 | 18.95 | 1.74 | 0.47 |
| C27 | 2.42 | 1.36 | 3.11 | 15.00 | 3.28 | 0.90 | 14.15 | 5.68 | 56.89 | 6.80 | 55.12 | 2.11 | 4.51 | 0.96 | 3.95 | 26.00 | 1.50 | 0.42 |
| C28 | 3.39 | 1.69 | 8.06 | 20.48 | 3.02 | 5.95 | 18.88 | 5.27 | 57.82 | 7.20 | 49.83 | 6.02 | 6.76 | 1.18 | 5.43 | 14.05 | 1.25 | 0.48 |
| C29 | 2.84 | 1.62 | 6.42 | 15.39 | 2.96 | 0.99 | 16.30 | 0.13 | 72.37 | 10.52 | 40.12 | 5.57 | 3.47 | 1.83 | 8.40 | 14.74 | 1.49 | 0.75 |
| C30 | 3.50 | 1.61 | 5.47 | 15.87 | 2.28 | 6.83 | 18.51 | 0.27 | 68.41 | 5.27 | 39.82 | 8.97 | 10.84 | 1.68 | 5.35 | 21.05 | 2.23 | 1.36 |
| C31 | 4.15 | 1.51 | 4.94 | 17.62 | 3.04 | 7.29 | 18.17 | 5.82 | 65.65 | 5.18 | 49.82 | 5.88 | 7.51 | 1.66 | 7.22 | 17.84 | 2.75 | 0.30 |
| C32 | 4.22 | 2.65 | 5.53 | 20.35 | 5.97 | 1.12 | 23.69 | 5.63 | 71.75 | 8.70 | 42.25 | 6.83 | 18.49 | 1.63 | 6.16 | 17.05 | 1.62 | 0.46 |
| C33 | 2.52 | 0.63 | 4.40 | 11.99 | 2.17 | 2.07 | 17.16 | 0.59 | 54.62 | 2.78 | 49.11 | 6.13 | 1.51 | 1.30 | 6.53 | 22.72 | 1.94 | 0.43 |
| A25 | 4.97 | 0.80 | 8.21 | 14.78 | 6.81 | 0.00 | 20.37 | 0.00 | 70.91 | 5.58 | 57.69 | 17.91 | 0.80 | 1.27 | 5.68 | 17.48 | 1.38 | 1.40 |
| A26 | 4.47 | 2.08 | 6.79 | 17.10 | 3.43 | 5.34 | 17.05 | 0.00 | 82.54 | 11.16 | 46.38 | 10.24 | 4.10 | 2.03 | 4.76 | 15.37 | 1.57 | 1.62 |
| A27 | 5.85 | 1.56 | 6.94 | 19.33 | 3.45 | 7.92 | 19.37 | 0.00 | 73.47 | 5.99 | 50.62 | 6.09 | 3.52 | 1.03 | 6.36 | 12.61 | 0.89 | 4.04 |
| A28 | 3.02 | 0.76 | 5.64 | 10.90 | 2.83 | 8.37 | 15.16 | 0.02 | 68.73 | 11.55 | 49.28 | 3.90 | 0.71 | 1.48 | 4.72 | 18.42 | 2.06 | 1.17 |
| A29 | 4.90 | 0.71 | 6.21 | 9.87 | 3.03 | 0.00 | 12.26 | 0.00 | 50.39 | 4.38 | 55.71 | 7.57 | 0.20 | 1.22 | 5.98 | 24.68 | 1.31 | 0.95 |
| A30 | 4.71 | 1.09 | 6.29 | 15.60 | 6.63 | 0.00 | 17.46 | 0.34 | 58.89 | 4.60 | 57.36 | 5.59 | 1.57 | 1.00 | 5.55 | 27.19 | 2.09 | 0.54 |
| A31 | 3.75 | 0.76 | 5.96 | 15.90 | 2.93 | 1.71 | 17.54 | 1.17 | 44.33 | 3.95 | 55.60 | 4.62 | 1.55 | 0.84 | 5.62 | 18.80 | 2.30 | 1.31 |
| A32 | 6.01 | 1.54 | 10.32 | 15.04 | 2.55 | 0.00 | 22.27 | 0.68 | 63.42 | 4.26 | 68.94 | 7.02 | 0.00 | 1.47 | 7.55 | 18.90 | 1.09 | 1.54 |
| A33 | 5.50 | 2.29 | 8.01 | 19.76 | 3.59 | 0.82 | 19.77 | 0.00 | 66.96 | 4.83 | 53.04 | 8.06 | 11.38 | 0.98 | 6.12 | 16.25 | 1.42 | 1.80 |
| A34 | 5.43 | 1.34 | 7.23 | 19.39 | 4.72 | 3.15 | 20.05 | 0.12 | 66.56 | 5.34 | 50.39 | 11.83 | 4.11 | 2.60 | 6.71 | 13.33 | 1.24 | 1.55 |

*TPM values for the LASSO-selected classifier transcripts measured in the 20 samples of the testing dataset.
