# Supplementary material for: Whole blood transcriptome biomarkers of unruptured intracranial aneurysm
Source: PLoS One. 2020 Nov 6;15(11):e0241838. doi: 10.1371/journal.pone.0241838 (PMC7647097; doi:10.1371/journal.pone.0241838)
Supplement: Attachment — Submitted filename: Response_to_Reviewers.docx [file pone.0241838.s007.docx]

October 01, 2020

**Authors response to reviewer comments for Manuscript PONE-D-20-25156, entitled “Whole blood transcriptome biomarkers of unruptured intracranial aneurysms”:**

Authors: We thank the editors and reviewers for their insightful comments, which have helped to improve the manuscript. On the basis of these comments, we have made several changes, which are tracked by yellow highlighting in the revised manuscript. Below are our point-by-point responses to the comments. The page numbers mentioned correlate with the revised version of the manuscript. Thank you.

**Editorial Comments (from 09/16/2020):**

***Journal Requirements:***

**1. Please ensure that your manuscript meets PLOS ONE's style requirements, including those for file naming.**

Authors: We have ensured that our manuscript meets PLOS ONE’s style requirements.

**2. Thank you for stating the following [not shown] in the Competing Interests section. Please confirm that this does not alter your adherence to all PLOS ONE policies on sharing data and materials, by including the following statement: "This does not alter our adherence to PLOS ONE policies on sharing data and materials.” (as detailed online in our guide for authors http://journals.plos.org/plosone/s/competing-interests). If there are restrictions on sharing of data and/or materials, please state these. Please note that we cannot proceed with consideration of your article until this information has been declared. Please include your updated Competing Interests statement in your cover letter; we will change the online submission form on your behalf.**

Authors: The conflicts of interest do not alter our adherence to all PLOS ONE policies on sharing data and materials. We have included the Competing Interests statement in the cover letter and added the following language:

"This does not alter our adherence to PLOS ONE policies on sharing data and materials.”

**3. We note that you have indicated that data from this study are available upon request. PLOS only allows data to be available upon request if there are legal or ethical restrictions on sharing data publicly. In your revised cover letter, please address the following prompts: a) If there are ethical or legal restrictions on sharing a de-identified data set, please explain them in detail (e.g., data contain potentially sensitive information, data are owned by a third-party organization, etc.) and who has imposed them (e.g., an ethics committee). Please also provide contact information for a data access committee, ethics committee, or other institutional body to which data requests may be sent. b) If there are no restrictions, please upload the minimal anonymized data set necessary to replicate your study findings as either Supporting Information files or to a stable, public repository and provide us with the relevant URLs, DOIs, or accession numbers. For a list of acceptable repositories, please see http://journals.plos.org/plosone/s/data-availability#loc-recommended-repositories.**

Authors: We have worked to be able to make the data publicly available. For the sequencing data in the discovery cohort (n=47, training dataset), we have uploaded the underlying participant-level data to NCBI’s Gene Expression Omnibus (GEO) under accession no. GSE159610. This includes deidentified raw RNA sequencing data, processed expression data, and patient metadata. For the validation cohort (n=20, testing dataset), we have made all expression levels for the LASSO-selected classifier transcripts used in this study available in their entirety in a new supplemental table, S6 Table. Therefore, we have changed our data availability statement

“Raw next-generation RNA sequencing data files and tables of processed transcript expression levels for the 47 samples in the training cohort in this publication can be found at NCBI’s GEO (accession no. GSE159610). All expression levels for the LASSO-selected classifier transcripts measured in the 20 samples of the testing dataset are presented in their entirety in S6 Table.”

This statement has been added to the cover letter. We have also added this information to the revised manuscript on page 12, under “Availability of RNA expression data”.

**Editorial Comments (from 10/09/2020):**

***Additional Comment:***

**1) We note that your Data Availability Statement indicates that some of the data used for this study may be proprietary data. Please be aware that PLOS journals will not consider manuscripts for which the conclusions depend solely on the analysis of proprietary data. We consider proprietary data to be data owned by individuals, organizations, funders, institutions, commercial interests, or other parties that the data owners will not share (as stated in https://journals.plos.org/plosone/s/data-availability#loc-unacceptable-data-access-restrictions). Therefore, can you please confirm whether other researchers would be able to obtain the data in the same manner by which the authors did?**

Authors: While other researchers would have been able to access the study data in the same manner as we did, we were able to ultimately make the data publicly available. See our modified response to question 3 above. Thank you.

**Reviewer Comments (from 09/16/2020):**

***Reviewer 1:***

**1. Proofreading. One example: "Currently, the only way to diagnose IAs is with cerebral imaging such as MR angiographjy, 59 computed tomographgy angiography (CTA), or digital subtraction angriography (DSA)." This is easily solved and does not affect either understanding or deliberation.**

Authors: Thank you for pointing this out. We have gone through the entire manuscript to correct spelling errors and typos throughout.

**2. Please include in the discussion what differences in signal occur testing peripheral blood compared to blood taken from an intracranial catheter during DSA.**

Authors: This is a good suggestion. Intuitively, we suspect that blood closer to the aneurysm may give greater signal in the biomarker. Differences in truly IA-associated transcripts would likely be exaggerated in blood samples drawn closer to the aneurysm tissue because the circulating cells that confer transcriptomic changes due to a blood-IA interaction would be most concentrated there. This is now discussed in the Discussion on page 15 of the revised manuscript.

**3. Mention that DSA is the gold standard and that CTA/MRA is less sensitive and what affect that may have, please.**

Authors: Thank you for this suggestion. In the limitations section of the Discussion on page 15 we now have stated that DSA is the gold standard, and that in future studies the use of CTA or MR angiography (which may be less sensitive to IA presence) to diagnose IAs could unintentionally give false positives for the proposed biomarker.

**4. Femoral access is done with a sheath, not a true catheter.**

Authors: Thank you for this comment. We have now corrected this in the Methods on page 4 of the revised manuscript.

***Reviewer 2:***

**1. Were the authors blinded when they performed the analysis on the tested group (not controlled/trained)? If not, then this MUST be done first before establishing the sensitivity, specificity, accuracy, PPV & NPV?**

Authors: Thank you for this question. Yes. The operators who input the data into the machine learning models were blinded to the class of the subjects. Only after the predictions were made, did the operator check the prediction with the true aneurysm status (yes or no) of the patient. This is now detailed in the Methods on page 7 of the revised manuscript.
